# Resveratrol as a Pan-HDAC Inhibitor Alters the Acetylation Status of Jistone Proteins in Human-Derived Hepatoblastoma Cells

**DOI:** 10.1371/journal.pone.0073097

**Published:** 2013-08-30

**Authors:** Sascha Venturelli, Alexander Berger, Alexander Böcker, Christian Busch, Timo Weiland, Seema Noor, Christian Leischner, Sabine Schleicher, Mascha Mayer, Thomas S. Weiss, Stephan C. Bischoff, Ulrich M. Lauer, Michael Bitzer

**Affiliations:** 1 Department of Internal Medicine I, Medical University Hospital, Tuebingen, Germany; 2 Evotec AG, Hamburg, Germany; 3 Section of Dermato-Oncology, Department of Dermatology and Allergology, University of Tuebingen, Tuebingen, Germany; 4 University Children's Hospital, Department of Hematology/Oncology, University of Tuebingen, Tuebingen, Germany; 5 Department of Nutritional Medicine, University of Hohenheim, Stuttgart, Germany; 6 Center for Liver Cell Research, Department of Pediatrics and Adolescent Medicine, University of Regensburg Hospital, Regensburg, Germany; Goethe University, Germany

## Abstract

The polyphenolic alcohol resveratrol has demonstrated promising activities for the prevention and treatment of cancer. Different modes of action have been described for resveratrol including the activation of sirtuins, which represent the class III histone deacetylases (HDACs). However, little is known about the activity of resveratrol on the classical HDACs of class I, II and IV, although these classes are involved in cancer development or progression and inhibitors of HDACs (HDACi) are currently under investigation as promising novel anticancer drugs. We could show by *in silico* docking studies that resveratrol has the chemical structure to inhibit the activity of different human HDAC enzymes. *In vitro* analyses of overall HDAC inhibition and a detailed HDAC profiling showed that resveratrol inhibited all eleven human HDACs of class I, II and IV in a dose-dependent manner. Transferring this molecular mechanism into cancer therapy strategies, resveratrol treatment was analyzed on solid tumor cell lines. Despite the fact that hepatocellular carcinoma (HCC) is known to be particularly resistant against conventional chemotherapeutics, treatment of HCC with established HDACi already has shown promising results. Testing of resveratrol on hepatoma cell lines HepG2, Hep3B and HuH7 revealed a dose-dependent antiproliferative effect on all cell lines. Interestingly, only for HepG2 cells a specific inhibition of HDACs and in turn a histone hyperacetylation caused by resveratrol was detected. Additional testing of human blood samples demonstrated a HDACi activity by resveratrol *ex vivo*. Concluding toxicity studies showed that primary human hepatocytes tolerated resveratrol, whereas *in vivo* chicken embryotoxicity assays demonstrated severe toxicity at high concentrations. Taken together, this novel pan-HDACi activity opens up a new perspective of resveratrol for cancer therapy alone or in combination with other chemotherapeutics. Moreover, resveratrol may serve as a lead structure for chemical optimization of bioavailability, pharmacology or HDAC inhibition.

## Introduction

Resveratrol (3,5,4′-trihydrostilbene) is a natural polyphenolic alcohol (Figure S1 in File S1) expressed in plants as response to external stress, like UV irradiation, fungal infection or injury [1]. The highest concentrations of resveratrol were detected in red grapes (100 µg/g) [2]. Therefore wine, especially red wine, contains concentrations of resveratrol between 0.2 mg/l to 7.7 mg/l [3], [4].

Resveratrol has attracted attention in the past years as it is assumed that consumption of red wine and thus the uptake of resveratrol are correlated with a low incidence of heart diseases despite of a saturated fat rich diet [5], [6]. Beside the protection from cardiovascular diseases [7] and antioxidant properties [8] resveratrol was described to possess antiinflammatory [9] and antiproliferative effects [10], [11]. These diverse modes of action are mainly driven by modulations of important intracellular proteins like NF-kB, p53, survivin, Bcl2 and the sirtuin SIRT1 [12]–[14]. Due to its multiple molecular interactions, resveratrol was analyzed for the treatment of cancer and identified to inhibit initiation and/or progression of several tumor entities like leukaemia [15]–[17], breast cancer [18], colon cancer [19], pancreatic cancer [20], gastric cancer [21], prostate cancer [22], lung cancer [23], melanoma [24] and tumors of the liver [25], [26].

In the last years, epigenetic modulation, especially modification of DNA-associated histone proteins received attention as new targets for cancer treatment. Regarding the modifications of histone proteins, changes of the acetylation status are most pronounced. Two antagonistic enzyme families govern histone acetylation: histone acetyltransferases (HATs) are involved in the acetylation of histone proteins, whereas histone deacetylases (HDACs) remove these acetyl groups from histone proteins [27]–[29]. Deacetylation of histone proteins by HDACs results in a more condensed chromatin structure and thus constricts the transcription of the DNA. HATs are the antagonistic enzyme family of HDACs and cause a relaxation of the chromatin structure [30]. For different cancer types a disarranged acetylation pattern of histone proteins caused by an altered recruitment and expression of HDACs was reported. The imbalanced equilibrium of HDACs and HATs changes gene expression [31] and is associated with tumor development and progression [28].

For human cells 18 different HDAC isoenzymes were described [28], [29]. These HDACs were subdivided into four different classes according to their cellular localization and homology to yeast. HDAC class I, II and IV are regarded as the classical HDAC enzyme families, while class III consists of sirtuins, a conserved and NAD^+^-dependent HDAC family. Targeting HDAC class I, II and IV by specific inhibitors has become a new promising approach for the treatment of cancer. Today, only the two HDAC inhibitors (HDACi) suberoylanilide hydroxamic acid (SAHA, Vorinostat™) and the microbial metabolite FK228 (Romidepsin, Istodax™) have been approved by the FDA for the treatment of cancer [32], [33]. Therefore, there is an unmet need for new HDACi compounds and especially for HDAC isoenzyme specific substances with a favourable safety profile in cancer drug development.

Natural products and compounds like resveratrol exhibit several biological functions [14]. Interestingly, resveratrol was identified as activator of the conserved HDAC class III family of the sirtuins [13], [14], [34]. We in turn were interested in a modulation of classical HDAC enzymes of class I, II and IV by resveratrol, due to earlier reports indicating that despite the well described activation of sirtuins, resveratrol might also inhibit HDACs [13], [34], [35]. Moreover, we intended to investigate whether the known antiproliferative effect of resveratrol on solid tumor cells of hepatocellular carcinoma (HCC) origin could be linked to the proposed epigenetic modulation of classical HDAC enzymes.

Detailed *in silico* and *in vitro* analyses demonstrated that resveratrol has the ability to inhibit human class I, II and IV HDACs. Moreover, for the hepatoma cell line HepG2 a specific inhibition of these enzyme families and consequently a hyperacetylation of the histone proteins was detected in the course of treatment with resveratrol. Incubation of human blood with high doses of resveratrol illustrated the Inhibition of HDACs in primary human cells. Of note, concentrations that exerted HDACi activity were well tolerated *in vitro* and *in vivo* by non-malignant cells.

## Materials and Methods

### Ethics Statement

Primary human hepatocytes (PHH) from different donors were provided via the charitable state-controlled foundation Human Tissue & Cell Research HTCR (http://www.htcr.de) with written informed patient's consent approved by the local ethical committee of the University of Regensburg, Germany [36], [37]. Blood samples for this study were obtained with written informed volunteeŕs consent for which the local ethical committee of the Faculty of Medicine, University of Tuebingen, Germany waived the need for further approval. All experiments involving human tissues and cells have been carried out in accordance The Code of Ethics of the World Medical Association (Declaration of Helsinki).

### Cell Culture and Reagents

Human-derived cancer cell lines HepG2, Hep3B, HuH7 were obtained from the German Collection of Microorganisms and Cell Cultures (DSMZ, Braunschweig, Germany) and cultured in DMEM with 10% fetal calf serum (FCS) and 2 mmol/l L-glutamine (Life Technologies, Rockville, MD, USA). PHH were cultured in DMEM with 100 U/ml penicillin and streptomycin (Serva, Heidelberg, Germany), 2 mmol/l L-glutamine, 18.8 µg/ml hydrocortisone (Merck, Darmstadt, Germany) and 1.68 mU/ml insulin (Novo Nordisk, Bagsvaerd, Denmark). Resveratrol was obtained from Sigma-Aldrich (>99% purity, CAS: R5010, Sigma-Aldrich, Taufkirchen, Germany) and solved in DMSO (CAS: D5879, Sigma-Aldrich). Suberoylanilide Hydroxamic Acid (SAHA) was obtained from IBL Hamburg (>98% purity, CAS: CM10009929, IBL Hamburg, Hamburg, Germany).

### Docking Analysis

Docking analysis was performed with human HDAC2, 4, 7 and 8 with resveratrol (Figure S1 in File S1) and the two reference HDACi compounds SAHA and trichostatin A (TSA). Due to the acidity of the phenol groups present in resveratrol the non-ionized and the different de-protonated isoforms were considered. All ligands were prepared using the molecular operation environment (MOE, version 2009.10, Chemical Computing Group Inc, Montreal, Canada). 3D representations of the ligands were obtained by energy minimization (Rebuild3D function with preservation of existing chiral centers) using MM94x force field and a Born Solvation model without cutoff constraints. All other parameters were left at default.

Crystal structures of HDAC2 (PDB code: 3max), HDAC4 (PDB code: 2vqm), HDAC7 (PDB code: 3c10) and HDAC8 (PDB code: 1t64) were retrieved from the protein data bank (PDB, http://www.ebi.ac.uk/pdbe/) and loaded into MOE. The Protonate3D functionality was applied to assign the correct ionization state and geometries to the protein atoms and to add hydrogen atoms. For the final docking water molecules were discarded. Docking was performed using GOLD (version 4.1.2, The Cambridge Crystallographic Data Center, Cambridge, UK). No additional protein preparation was applied. Binding sites were defined by all residues within 5 Å distance from the corresponding ligands in the crystal structure. Docking was performed using GoldScore as scoring function. All other parameters were left at default. Docking was validated by comparing the highest scoring docking pose of TSA to the pose of the ligand in the corresponding crystal structure of HDAC7 and HDAC8. In both cases excellent overlays were obtained with RMSDs below 1.5 Å. Docking poses of resveratrol in the individual HDAC binding pockets were analyzed in MOE. To optimize the ligand-receptor interaction an energy minimization was applied using MM94x force field and a Born Solvation model without cutoff constraints.

### HDAC Inhibitor Screening Assay

HDAC inhibitor screening was done with the HDAC assay kit (Active Motif, La Hulpe, Belgium). As HDAC source HeLa nuclear extract (Active Motif) was used. Assay was performed as described by the manufacturer except changes in the incubation time (37°C for 2 h) and developing time (10 min).

For HepG2 HDAC inhibition determination, nuclear extract of HepG2 cells (Active Motif) was used. The extract was diluted with HDAC assay buffer (Active Motif), so that a final concentration of 5 µg/well was achieved. Assay was performed according to manufactureŕs protocol except changes in the incubation time (37°C for 2 h) and developing time (10 min).

To exclude a possible disturbance by the autofluorescence of resveratrol in these assays an additional background control with 100 µM resveratrol was performed. No interference was detected in our experimental setting.

### HDAC Inhibition Profiling Assay

The human HDAC profiling assay was performed on basis of the *Fluor de Lys*™ technology by Scottish Biomedical, Glasgow, UK. The percentage inhibition values of 50 µM and 100 µM resveratrol against human HDAC enzymes HDAC1, HDAC2, HDAC3, HDAC4, HDAC5, HDAC6, HDAC7, HDAC8, HDAC9, HDAC10 and HDAC11 were determined. All assays were performed in 1% DMSO (final concentration).

### Real-time Cell Proliferation Assay

HepG2 (10^4^ cells/well), Hep3B (5×10^3^ cells/well) and HuH7 (2.5×10^3^ cells/well) were seeded in 96-well plates (E-Plate 96, Roche Applied Science, Mannheim, Germany). Real-time dynamic cell proliferation was monitored in 30 min intervals over 106 h using the xCELLigence SP system (Roche Applied Science). Cell index values were calculated using the RTCA Software (1.2.1.1002). All curves were normalized at the beginning of the treatment period (10 h after seeding) applying the RTCA Software.

### IC_50_ Determination

IC_50_ values of resveratrol were determined for the different cell lines according to manufacturer’s protocol with RTCA Software (Version 1.2.1.1002, Roche Applied Science).

### Sulforhodamine B (SRB) Assay

HepG2, Hep3B and HuH7 cells (2×10^4^ cells/well) or PHH (3×10^5^ cells/well) were seeded in 24-well plates. Growth inhibition was evaluated at dedicated time points by SRB assay. Assay was performed as described [38]; in brief: cells were fixed by incubation with 10% trichloroacetic acid for 30 min after washing twice with PBS. After drying at 40°C, cells were stained with sulforhodamine B (SRB, CAS: S9012, Sigma-Aldrich), unbound dye was carefully removed by washing and the cells were dryed for 3–6 h at 40°C. Cell-bound SRB was washed out with 10 mM Tris (pH 10.5) and optical density was measured with GENios Plus (TECAN, Crailsheim, Germany). Data represent the mean of optical density measurements related to untreated control cells.

### Acetylation Assay

HepG2, Hep3B (3×10^4^ cells/well) and HuH7 (10^4^ cells/well) were seeded in a 96-well plate and incubated overnight. For the SIRT1 inhibition experiments EX527 was added to the cells 1 h before resveratrol treatment started. After dedicated time points, treated cells were fixed by incubating for 10 min in 150 µl 95% methanol at room temperature. Cellular Histone Acetylation Assay was performed according to manufactureŕs protocol (CAS: CY-1140, Cyclex Ltd., Nagano, Japan). Acetylation of intracellular proteins was detected via colorimetric measuring with GENios Plus (TECAN).

### Immunoblotting

HepG2, Hep3B and HuH7 (2×10^5^ cells/well) were seeded into 6-well plates, washed once (PBS), harvested and resuspended in 100 µl lysing-buffer (1% Nonident P40, 0.5 M Tris-Base (pH 7.6), 0.15 M NaCl). Lysates were stored at −80°C, thawed and refrozen 3 times and treated with sonifier (60% output volume, 20 sec). Cellular proteins were separated on 12% SDS-polyacrylamide gels under reducing conditions and transferred to polyvinylidene difluoride membranes (Hybond-P, Amersham Biosciences, Piscataway, NJ). Membranes were blocked in Tris-buffered saline (150 mmol/l NaCl, 13 mmol/l Tris, pH 7.5) containing 5% non-fat dry milk powder (1 h). Next, the membranes were incubated with anti-vinculin (1∶5,000, CAS: V 9131, Sigma-Aldrich, Germany) or anti-acetyl-histone H3 (1∶8,000, CAS: 06-599, Millipore, USA) overnight at 4°C, then washed three times with TBS-T (TBS containing 0.02% Triton X-100) and incubated with peroxidase-conjugated anti-rabbit (1∶8,000, Bio Rad, USA) or anti-mouse (1∶4,000, Bio Rad) for 45 min. Membranes were washed three times in TBS-T and further detection was performed by the ECL Western blotting detection system on Hyperfilm-ECL (Amersham Biosciences).

### HDAC Activity Determination in Human Blood

Blood samples were collected in citrate monovettes (Sarstedt, Nuembrecht, Germany). Anticoagulated blood was divided into 0.5 ml aliquots and incubated with 100 µM resveratrol for indicated time points at 37°C. To determine the background signal of every experiment 10 µM TSA were used. Then, HDAC substrate (Boc-Lys-AMC, Bachem, Bubendorf, Switzerland) was added to each sample (final concentration 100 µM) and incubated additionally for 2 h at 37°C. After incubation, whole sample was transferred into 2.5 ml of cooled erythrocyte lysis (EL) buffer (Qiagen, Hilden, Germany) and shook for 15 min at 4°C. The samples were centrifuged for 10 min, 400×g at 4°C, supernatant was removed and pellet resuspended in 1 ml EL buffer. After additional centrifugation with 400×g, 4°C for 10 min, supernatant was removed and cell pellet resuspended in 200 µl of a 1∶1 mixture of lysis (50 mM TRIS pH 8.0 (HCl), 137 mM NaCl, 2.7 mM KCl, 1 mM MgCl_2_, 1% NP-40 Alternative) and developing (50 mM TRIS pH 8.0 (HCl), 100 mM NaCl, 10 mg trypsine/ml) buffer. Suspension was transferred to black 96-well plate (Greiner-bio-one, Frickenhausen, Germany) with 100 µl/well, each sample in duplicate, incubated at 37°C for 20 min and fluorescence was measured at 450 nm (GENios Plus, TECAN).

### Lactate Dehydrogenase (LDH) and Aspartate Aminotransferase (AST) Assays

PHH (3×10^5^ cells/well) were seeded in 24-well plates. LDH concentration in the supernatant was determined with LDH-P mono kit (Biocon™, Voehl/Marienhagen, Germany) and AST with GOT-AST mono kit (Biocon™) as suggested by the manufacturer; all values are referred to untreated control cells (% control).

### Embryotoxicity Assay

Since chick embryos were used in very early stages, no ethical approval was required according to local animal care guidelines. Fertilized eggs of leghorn chickens (*Gallus Gallus domesticus*) were obtained from a hatchery (Weiss, Kilchberg, Germany) and incubated at 38°C in a temperature-controlled brooder (BRUJA Type 400a, Brutmaschinen Janeschitz, Hammelburg, Germany). For embryotoxicity testing, eggs were used after 50 h of incubation (equal to stage 13 according to Hamburger and Hamilton [39]), which corresponds to approximately six human gestational weeks [40]. The eggs were prepared as described previously [41]. Resveratrol was applied in concentrations of 5, 10, 20, 50 (n = 8) and 100 µM (n = 3) on top of the blastoderm. The assay was performed in two different experiments on different experimental days. Eggs treated with the solvent (ethanol) alone served as negative control. The eggs were sealed with adhesive tape and replaced into the incubator. 24 h, 48 h and 72 h after application of resveratrol, viability of the embryos was determined by verification of heart actions and blood flow in the chorioallantoic vessels. Survival rates were depicted as Kaplan-Meier curves.

### Statistical Analysis

Statistical analyses for different assays were performed with either Students t-test or with One-way ANOVA Dunnetts multiple comparison test using GraphPad Prism version 4.00 (GraphPad Software, San Diego, CA, USA). According to One-way ANOVA Dunnetts multiple comparison test all resveratrol treatment groups were compared vs. vehicle/control. All values of *P*<0.01 were defined as statistically significant and highlighted with *.

## Results

### *In silico* Docking Analyses Reveal an Inhibitory Potential of Resveratrol on Human HDAC Enzymes of Class I and II

**Figure 1:**
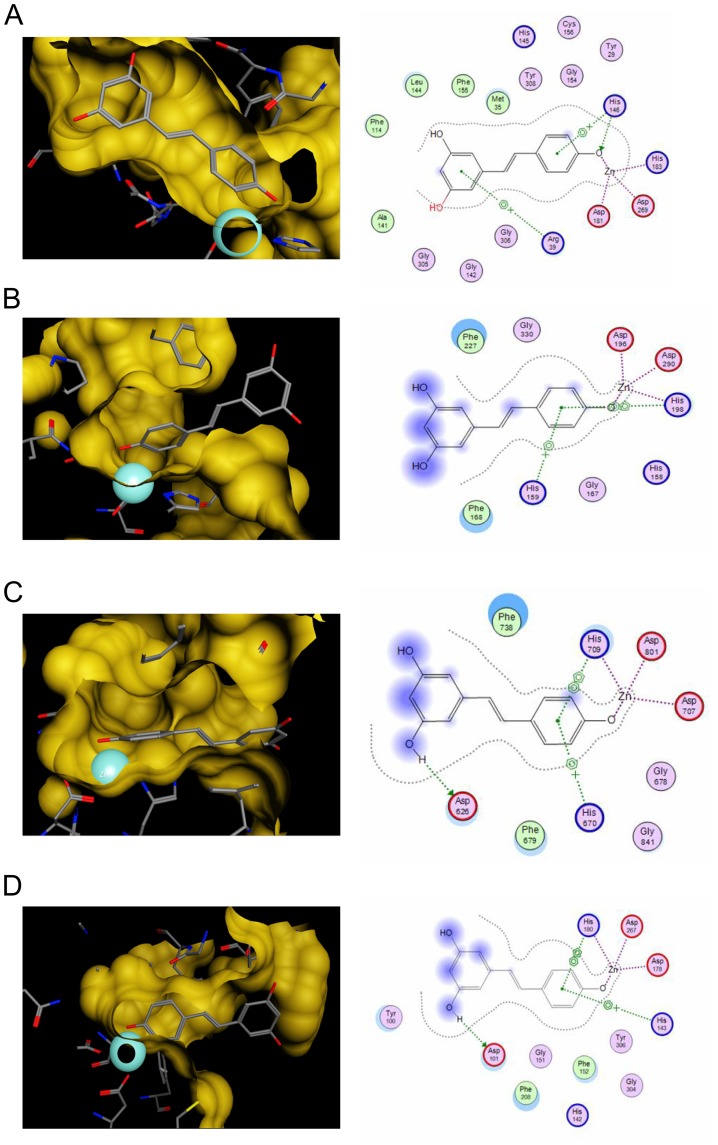
*In silico* docking analysis with resveratrol illustrates an inhibitory potential for human HDAC enzymes of classes I and II. (A–D) Results of the *in silico* docking analysis of resveratrol with crystal structures of HDAC2 (A), HDAC4 (B), HDAC7 (C) and HDAC8 (D). The analysis demonstrates the predicted binding mode of resveratrol in the different HDAC binding pockets. The predicted interactions of resveratrol with the zinc ion (turquoise sphere) and other residues of the catalytic center are highlighted in the left row. 2D depiction of resveratrol along with interacting amino acids is shown in the right row. Green circles represent hydrophobic, purple circles polar, red circles acidic and blue circles basic residues. Blue halolike discs around amino acids are calculated based on the reduction of solvent exposure by the ligand. Blue arrows represent backbone H-bond interactions, green ones depict sidechain H-bond interactions. Green benzol rings with a “+” describe arene-cation interaction, 2 benzol rings an arene-arene interaction. Areas with a blue background are solvent exposed parts of the ligand. The purple dotted lines represent metal contact.

To analyze a putative inhibition of different HDAC enzymes *in silico*, docking analyses of resveratrol with members of HDAC class I (HDAC2, HDAC8) and class II (HDAC4, HDAC7) were performed (Figure 1A–D). Unfortunately, protein data allowing *in silico* docking of class IV HDAC enzymes are not yet available. Important features for the identification of a HDACi are both the structural property to fit into the binding pocket of HDAC enzymes and the capacity to interact with key interaction points like the zinc ion in the catalytic center. Docking results with HDAC2, 4, 7 and 8 displayed that both crucial requirements were fulfilled. Resveratrol was able to fit into the binding pocket of all tested HDACs and interacted with the zinc ion as well as with amino acid residues of the catalytic pocket (Figure 1A–D).

As positive controls, we employed recently published *in silico* data by Berger et al. of the well characterised HDACi trichostatin A (TSA) and the clinically used HDACi SAHA [42]. A direct comparison between the established HDACi TSA, SAHA, and resveratrol was achieved by calculating the binding affinity using the scoring function GoldScore (Table S1 in File S1). GoldScore values of resveratrol were situated between those of SAHA, which had the highest values, and TSA. These data led to the assumption that resveratrol substantially inhibits at least class I and II HDACs.

### *In vitro* Inhibitor Analyses Reveal a pan-HDACi Activity of Resveratrol

**Figure 2:**
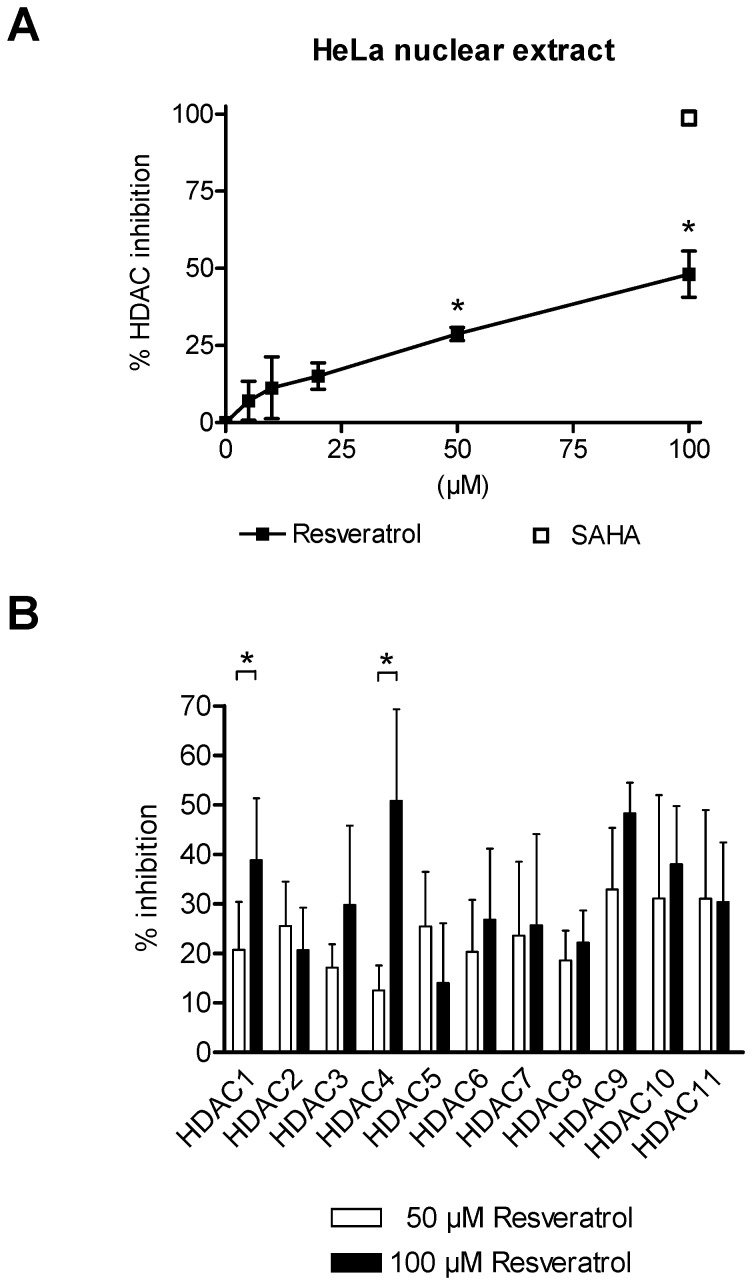
HDAC inhibition mediated by resveratrol. (A) Overall inhibition of human HDAC enzymes in HeLa nuclear extracts by increasing concentrations of resveratrol (5 µM, 10 µM, 20 µM, 50 µM and 100 µM). As a reference inhibitor suberoylanilide hydroxamic acid (SAHA; 100 µM) was used. Every concentration was tested three times in triplicates; One-way ANOVA Dunnetts multiple comparison test, *P<0.01. (B) Specific fluorometric profiling assay using recombinant human HDACs of classes I, II and IV. Specific inhibition values were generated for the treatment with 50 µM and 100 µM resveratrol. Inhibition values for every HDAC were yielded by four independent experiments, each performed in duplicates; Students t-test *P<0.01. Shown are mean ± SD (A and B).

Based on the *in silico* results, additional *in vitro* analyses were performed. As a first approach a HDAC inhibitor screening assay with 5 µM, 10 µM, 20 µM, 50 µM and 100 µM of resveratrol was performed to evaluate overall HDAC inhibition in a low concentration range (Figure 2A). In accordance with the *in silico* analysis, resveratrol showed inhibition activity of human HDAC enzymes expressed in nuclear cell extracts *in vitro*. Interestingly, significant inhibition values for 50 µM and 100 µM resveratrol were detected in comparison to the vehicle treated control (*P<0.01; Figure 2A).

To further substantiate the *in silico* and *in vitro* results and to itemise the inhibitory activity of resveratrol, an additional profiling assay was performed. On basis of the moderate HDACi activity in the HDAC inhibitor screening assay at low concentrations, only 50 µM and 100 µM of resveratrol were chosen for further testing. The profiling results affirmed the inhibition potential of resveratrol with differences in the activity for the eleven conserved human HDACs of class I, II and IV (Figure 2B). In line with our previous results, only moderate inhibition values for resveratrol were detected at 50 µM for HDAC1 up to HDAC11. Treatment with 100 µM resveratrol resulted in a slight increase of the inhibition values, being only statistically significant for HDAC1 and 4 (Figure 2B). Nevertheless, according to the different analyses, resveratrol can be classified as a moderate pan-HDACi.

### Antiproliferative Effect of Resveratrol on Human Hepatoma Cell Lines

Resveratrol has been described to exert antiproliferative effects on different tumor entities including HCC [25], [26]. To transfer the newfound pan-HDACi activity of resveratrol into a potential cancer treatment setting, we investigated the therapeutic potential of resveratrol on different human hepatoma cell lines.

**Figure 3:**
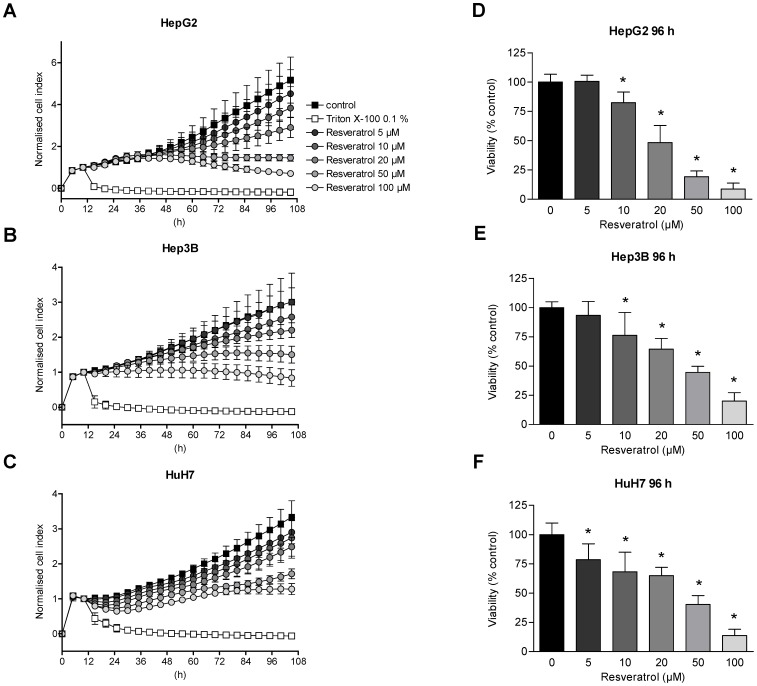
Reduced proliferation and viability of hepatoma cells by resveratrol. (A–C) HepG2 (A), Hep3B (B) and HuH7 (C) hepatoma tumor cells were treated with different concentrations of resveratrol (5 µM, 10 µM, 20 µM, 50 µM and 100 µM) or solvent as control and monitored for an additional time period of 96 h. Cellular impedance was measured over the entire observation time using the xCELLigence™ SP system and calculated by the RTCA Software 1.2.1.1002. All cell index (CI) values were normalized when treatment started. Displayed are normalized CI values every 5 h. As a positive control for cell death, Triton X-100 0.1% was used. (D–F) Sulforhodamine B (SRB) assay of HepG2 (D), Hep3B (E) and HuH7 (F) cells treated with increasing concentrations of resveratrol (5 µM, 10 µM, 20 µM, 50 µM and 100 µM) or solvent as control for 96 h. Shown are mean ± SD of three independent experiments, each performed in triplicates (A–F); One-way ANOVA Dunnetts multiple comparison test, *P<0.01 (D–F).

Proliferation and cellular viability of cell lines HepG2, Hep3B and HuH7 were monitored continuously over time using a real-time cell monitoring assay. Consistent with the HDAC inhibitor screening and profiling assays, the cells were treated once with increasing concentrations of resveratrol (Figure 3A–C). Measurement of the cellular impedance was depicted by the cell index (CI) and reflected the cellular status. Reduction of the CI can be used as surrogate marker for diminished cellular growth/proliferation or cytotoxic effects [43], [44]. As a result, the normalized CI showed a concentration-dependent decline after application of resveratrol in every hepatoma cell line tested (Figure 3A–C). However, we found a differential curve progression when HepG2 cells were compared with Hep3B and HuH7 cells. Especially within the first 24 h under resveratrol treatment, HepG2 cells initially show an increase of the CI, followed by a concentration-dependent decline, whereas Hep3B and HuH7 cells did not display a similar curve pattern prior to a CI decline, which is finally detected in all three cell lines (Figure 3A–C and Figure S2A–C in File S1). This distinct reaction pattern of HepG2 cells in the real-time cell proliferation assay hints to a different molecular response pattern to resveratrol in this cell line (Figure 3A–C and Figure S2A–C in File S1). Notably, these three hepatoma cells harbor well characterized molecular differences, e.g. HepG2 cells are known to be p53 wild-type, whereas Hep3B cells are p53 deficient due to a deletion of the p53 gene and HuH7 possess a mutated p53 gene [45], [46]. Nevertheless, the inhibitory concentrations that were calculated on the basis of the real-time proliferation assay (IC_50_ values) were quite similar after 96 h in all three cell lines (Table S2 in File S1). To further validate the antiproliferative potential of resveratrol on the different hepatoma cell lines, sulforhodamine B (SRB) assays were performed (Figure 3D–F). In line with the real-time experiments, HepG2, Hep3B and HuH7 cells were treated once with different concentrations of resveratrol and analyzed after 96 h of incubation. Overall protein synthesis, detected by the SRB assay confirmed the results of the real-time cell monitoring. Treatment of the different hepatoma cells with resveratrol for 96 h induced a similar concentration-dependent reduction of cell viability for every cell line tested (Figure 3D–F).

### Resveratrol Induces HDAC Inhibition and Consequently Hyperacetylation of Histone Protein H3 only in HepG2 Cells

**Figure 4:**
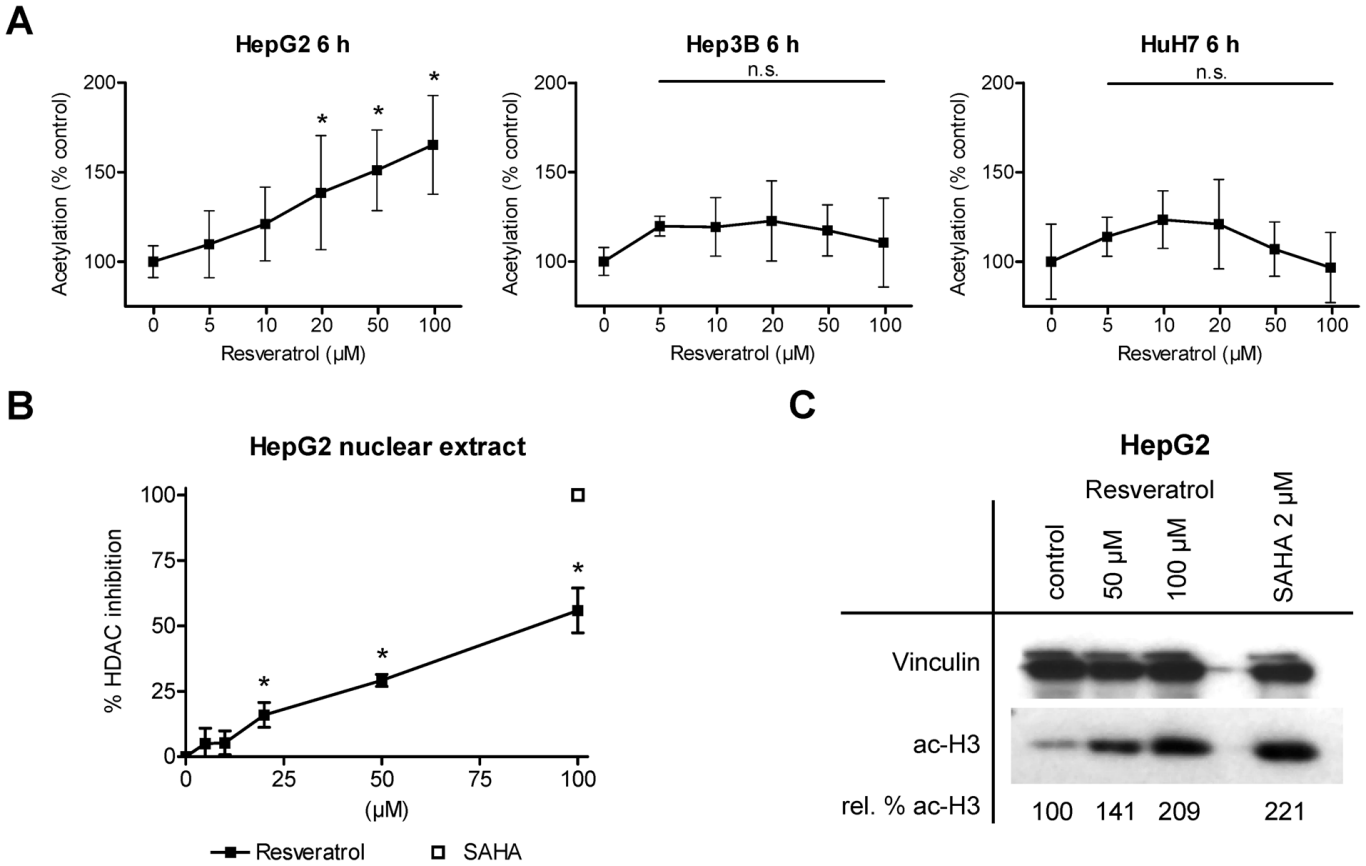
Resveratrol induces overall hyperacetylation in HepG2 but not Hep3B or HuH7 cells. (A) Detection of intracellular acetylated protein levels in HepG2, Hep3B and HuH7 hepatoma cells after incubation of resveratrol (5 µM, 10 µM, 20 µM, 50 µM and 100 µM) or solvent as control for 6 h. (B) Overall HDAC inhibition in nuclear extracts of HepG2 cells by increasing concentrations of resveratrol (5 µM, 10 µM, 20 µM, 50 µM and 100 µM) or solvent as control. As a reference inhibitor suberoylanilide hydroxamic acid (SAHA; 100 µM) was used. (C) Western blot analysis of acetylated histone complex H3 in HepG2 tumor cells treated with 50 µM and 100 µM of resveratrol or solvent as control. Acetylation of H3 was examined using cellular lysates. Equal protein loading was verified by vinculin staining (upper row). As a reference and positive control for hyperacetylation the cells were treated with 2 µM SAHA. Acetylation levels were calculated performing a densitometric analysis. Shown are mean ± SD of three independent experiments, each performed in triplicates (A and B); One-way ANOVA Dunnetts multiple comparison test, *P<0.01, n.s. indicates not significant (A and B).

Due to the newfound pan-HDACi activity and the profound antiproliferative effects, we analyzed the overall acetylation status of HepG2, Hep3B and HuH7 cells. Therefore, 6 h after applying resveratrol at various concentrations, the concentration of acetylated proteins was determined (Figure 4A). Of note, incubation with 20 µM, 50 µM and 100 µM resveratrol did induce a significant dose-dependent hyperacetylation in HepG2 cells (*P<0.01), whereas in Hep3B or HuH7 no change in the acetylation status was observed (Figure 4A). Also a prolonged incubation time of 24 h did not alter the results for Hep3B or HuH7 cells (Figure S3 in File S1).

To analyze the resveratrol-mediated hyperacetylation in HepG2 hepatoma cells a specific HepG2 HDAC inhibition screening assay (Figure 4B) and a HepG2 Western blot analysis of acetylated histone protein H3 (Figure 4C) were performed. In analogy to the standardized HDAC inhibitor screening assay we observed an inhibition activity for resveratrol in the specific HepG2 HDAC inhibition screening assay using nuclear extract of HepG2 cells. In line with previous results, we detected significant inhibition values for 20 µM, 50 µM and 100 µM resveratrol (*P<0.01; Figure 4B). Also the Western blot analysis revealed a prominent hyperacetylation of histone protein H3 after resveratrol treatment with 50 µM and 100 µM in HepG2 cells (Figure 4C). In contrast, but in line with the overall acetylation status (Figure 4A), Western blot experiments with extracts of Hep3B and HuH7 cells did not show any hyperacetylation after the treatment with 50 µM or 100 µM resveratrol (Figure S4 in File S1).

### HDAC Inhibition by Resveratrol in Human Blood

**Figure 5:**
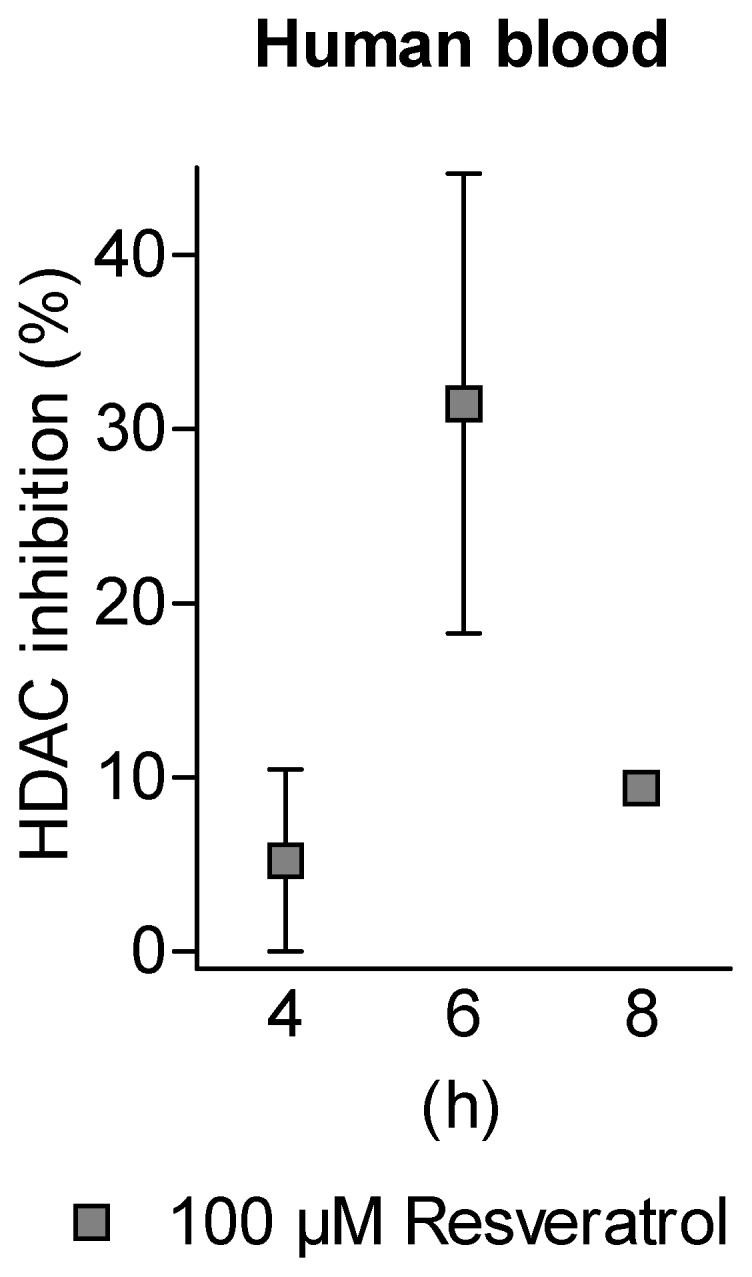
HDAC inhibition by resveratrol in human blood. Anticoagulated human blood was incubated with 100 µM of resveratrol for 4 h, 6 h and 8 h. HDAC activity of lysates of peripheral blood cells was measured *ex vivo*. Inhibition values of HDAC activity were yielded by three independent experiments, each performed in duplicates. Shown are mean ± SEM.

On basis of these results and to investigate whether resveratrol is able to modulate HDAC activity in primary human-derived cells, whole human blood was incubated for 4 h, 6 h or 8 h with the highest concentration of resveratrol and the inhibition of HDAC enzymes in the lysates of Peripheral Blood Mononuclear Cells (PBMCs) was determined. In line with the *in silico* and *in vitro* findings we observed a resveratrol mediated inhibition of HDACs in PBMCs, which was most prominent at 6 h of incubation and decreased afterwards (Figure 5). According to these results we conclude that resveratrol when present at high concentrations like 100 µM in the blood is able to enter human cells and modulate HDAC activity.

### Resveratrol Shows No Cytotoxic Side Effects on Non-malignant Cells *in vitro* but *in vivo*

**Figure 6:**
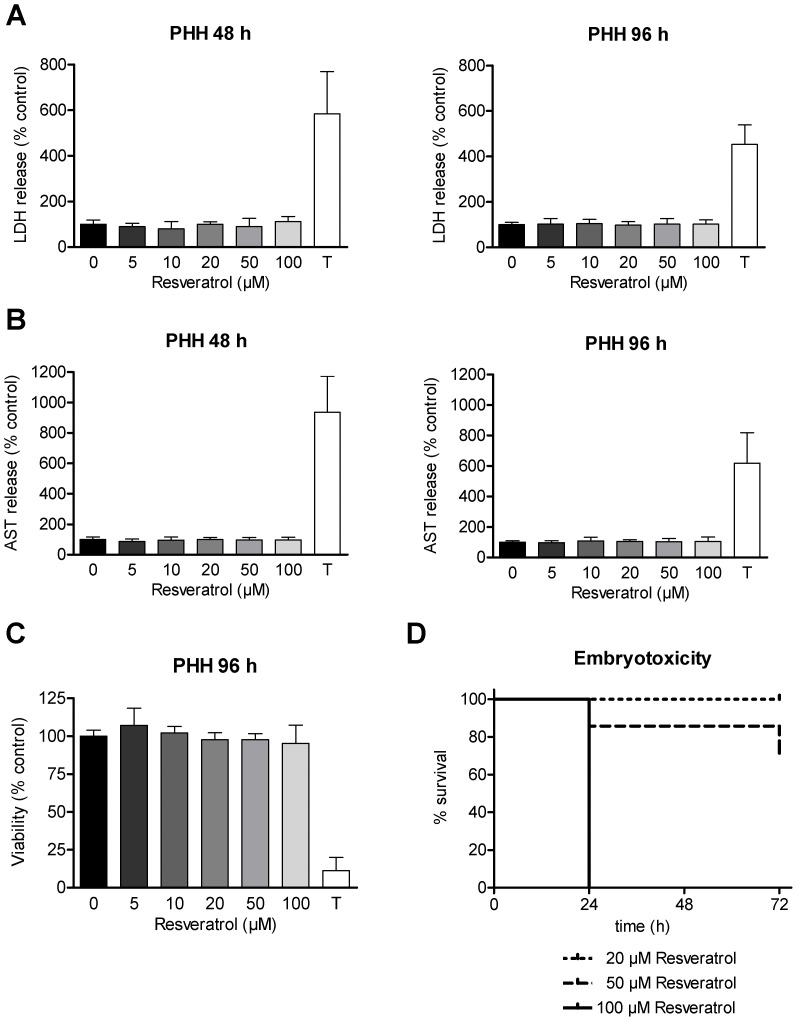
Toxicity profile of resveratrol in non-malignant cells. (A–C) Non-malignant primary human hepatocytes (PHH) from different donors were treated with increasing concentrations of resveratrol (5 µM, 10 µM, 20 µM, 50 µM and 100 µM) or solvent as control. Lactate dehydrogenase (LDH) release (A) and aspartate aminotransferase (AST) release (B) into the supernatant fluid were determined after 48 h and 96 h. Sulforhodamine B (SRB) assay (C) of PHH treated for 96 h. The incubation with Triton X-100 1% was performed as a positive control for cell death in all experiments. Bars represent mean ± SD of three independent experiments with PHH from three different human donors, each performed in triplicates (A–C). (D) Chicken embryotoxicity assay with different concentrations of resveratrol or solvent as control. Chicken embryos stage 13 (corresponding to 6 human gestational weeks) were exposed to rising concentrations of resveratrol to determine embryotoxic effects. Survival rates after 24 h, 48 h and 72 h are depicted in a Kaplan-Meier plot.

For any clinical application of resveratrol an in depth evaluation of toxicity with non-malignant cells is needed, employing concentrations that both are (i) effective in antiproliferation on tumor cells and simultaneously are (ii) epigenetically active. Therefore, PHH from three different human donors were treated with the same concentrations of resveratrol used for the hepatoma cell lines. As a marker of cellular membrane integrity LDH release into the supernatant was measured for 48 h and 96 h of incubation with resveratrol (Figure 6A). The LDH release assay showed no resveratrol-mediated cytotoxicity on PHH. Additionally, the liver specific enzyme AST was measured after 48 h and 96 h of resveratrol treatment (Figure 6B). In accordance with the results of the LDH release assay, no significant increase was observed for any concentration tested. To allow a direct comparison between tumor and non-malignant cells, a SRB assay was performed after 96 h of resveratrol treatment (Figure 6C). Consistent with the LDH and AST results, no signs of toxicity for resveratrol were observed. Taken together, resveratrol seemed to be well tolerated by PHH at concentrations that were found to induce a severe reduction of viability in different tumor cell lines.

Epigenetic drugs, especially HDACi, can cause embryotoxic effects [47]. Therefore, in addition to the evaluation of the cytotoxicity mediated by resveratrol in non-malignant hepatocytes, we performed an embryotoxicity assay. Chicken embryos were incubated with 5 µM, 10 µM, 20 µM, 50 µM or 100 µM of resveratrol and survival was monitored at 24 h, 48 h and 72 h (Figure 6D). For this experiment, resveratrol was dissolved in 50% ethanol to avoid unwanted side effects of the solvent, since DMSO is known to cause embryotoxic side effects (data not shown). In contrary to PHH, chicken embryos showed strong toxic effects under treatment with 100 µM resveratrol. In contrast, concentrations of 20 µM and below were tolerated well and exhibited no signs of toxicity (only 20 µM shown).

## Discussion

Cancer cells are characterized by various molecular changes. It is believed that especially epigenetic modifications play a crucial role in tumor development and maintenance [28]. In this context, the inhibition of HDAC enzymes has become an important therapeutic target and has shown promising results in cancer therapy. So far, only two HDACi are approved by the FDA for clinical use [32], [33]. Although some HDACi are currently tested in clinical trials, the identification of new inhibitors with low side effects has become of major interest. In recent years, a growing number of nutrients and dietary substances with chromatin modulating potential and epigenetic activity have been indentified [42], [48], [49].

The natural polyphenol resveratrol is a promising candidate for cancer treatment due to its proapoptotic and antiproliferative effects [14]. Resveratrol modulates multiple cellular signalling pathways and has demonstrated to be epigenetically active [14], [35], [50], [51]. Due to the pleiotropic biological activities of resveratrol, we were interested in a possible resveratrol-mediated modulation of the classical HDAC enzyme families I, II and IV, which play an eminent role in tumor formation and growth.

By *in silico* docking analysis we detected a resveratrol-mediated inhibition of HDAC enzymes of classes I and II. Structure-activity studies showed that resveratrol fits into the binding pocket of HDACs and interacts with the zinc ion as well as other amino acid residues forming the active site. As demonstrated by established HDACi like SAHA and TSA, binding and interaction with the catalytic center are important features for HDACi activity. The predicted inhibition potential was approved *in vitro* by a standardized HDAC inhibitor screening assay as well as profiling studies for all known classical human HDAC enzymes. According to our *in vitro* and *in silico* results we postulate that resveratrol is a pan-HDACi. The observed weak HDACi potential of resveratrol however was not surprising and is in line with the hypothesis that nutritional HDACi are expected to exert moderate inhibitory activities [52].

On the other hand, in mice resveratrol is described as an activator of SIRT1, a member of the mammalian sirtuins or HDAC class III, respectively. Furthermore, some of the resveratrol-mediated antitumor activity seems to be attached to the modulation of SIRT1 [53]. Therefore, it is rather expected that resveratrol induces a deacetylation instead of an acetylation of histone proteins [13], [14], [34]. But sirtuins differ drastically from classical HDAC enzymes, not only by depending on the co-factor NAD^+^, but also by an altered intracellular localization and substrate specificity [13]. Sirtuins display a highly conserved catalytic and NAD^+^-binding domain, which is often described as the sirtuin core domain and not found in HDACs of classes I, II or IV. The exact mechanism how resveratrol interacts with sirtuins has been under intense investigation during the last years. Besides a direct SIRT1 activation, evidence exists that resveratrol is also able to enhance the association between SIRT1 and other cellular factors that directly influence the activity of SIRT1 [54]–[56]. Nevertheless, due to the pleiotropic molecular mechanisms of resveratrol it is reasonable that it may activate sirtuins as well as inhibit HDACs of classes I, II and IV. Interestingly, an elegant recent study that investigated a model of wound repair revealed that resveratrol induced a downregulation of the DNA binding capacity and of the activity of HDAC2 via activation of sirtuins [57]. Inhibiting SIRT1 in our HepG2 tumor cell system prior to resveratrol treatment resulted in a slightly decreased hyperacetylation of cellular proteins, which did not reach significance (Supplementary Figure S5A). These data suggest that the cross-talk between SIRT1 and classical HDACs is rather weak in our test system and a direct HDAC inhibition by resveratrol occurs in HepG2 cells. Possible mechnisms how resveratrol could modulate HDAC activity are schematically summarized in Figure S5B in File S1.

To investigate the clinically interesting newly found pan-HDACi activity of resveratrol into a cancer treatment setting, we exemplarily used a hepatoma tumor model. As a first approach we analyzed HepG2, Hep3B and HuH7 cells after treatment with concentrations ranging from 5 µM up to 100 µM of resveratrol. Particularly the upper concentrations of 50 µM and 100 µM were chosen to ensure substantial HDACi activity. In addition to that, studies with rats show, that these values can be achieved as peak concentrations after high dose intravenous administration despite a rapid decrease and subsequent elimination [58], [59]. Real-time cell monitoring experiments and SRB assays affirmed the anticancer properties of resveratrol when used in this dosage. The calculated IC_50_ concentrations of our real-time cell monitoring of HepG2 and HuH7 cells were in line with data which have been published previously [60], [61]. Interestingly, treatment of HepG2 cells with resveratrol triggered a delayed antiproliferative response within the first 24 h indicating different modes of action in the hepatoma cell lines. Taken together, these experiments underline the therapeutically attractive antiproliferative effects of resveratrol on cancer cells described for various tumor entities [15]–[21].

To analyze the postulated connection between epigenetic modulation by inhibition of HDAC enzymes and antiproliferative effects on tumor cells mediated by resveratrol, we evaluated the amount of acetylated intracellular proteins after treatment with low concentrations of the newfound pan-HDACi resveratrol. Surprisingly, our results showed that due to resveratrol treatment an increase of acetylated proteins was detectable only in HepG2 cells. The epigenetic mode of action was confirmed by Western blot analysis and a specific HDAC inhibition screening assay for HepG2 cells, strongly indicating that the antiproliferative effect in this cell line might at least be partly based on HDAC inhibition. For Hep3B and HuH7 hepatoma cells no change in the overall acetylation status was detected at all measured time points. Also, specific Western blots for acetylated histone protein H3 did not imply a resveratrol-mediated hyperacetylation. Although Hep3B and HuH7 tumor cells showed a similar decline of viability after treatment with resveratrol, an inhibition of HDACs was not detectable. It is noteworthy that tumor cells show an imbalance in the regulation of HDACs, mostly due to an overexpression of one or more HDAC enzymes [28]. Upregulation of even a single HDAC enzyme can induce oncogenic cell transformation and tumorigenesis [62]. According to the expression and activity of the HDACs in different tumor entities or cell lines, resveratrol mediates different cellular responses. Our results indicate a profound inhibition of HDACs in HepG2 cells but not in Hep3B or HuH7 cells. Based on the multiple described modes of action of resveratrol other mechanisms might also influence the observed effects in the investigated cell lines [7]–[14], [61].

The detected inhibition of classical HDACs by resveratrol is in line with recently published data, demonstrating that resveratrol restored gene expression in the human spinal muscular atrophy type I fibroblast cell lines GM03813 and GM09677 by a proposed epigenetic mechanism [35]. Our findings now go far beyond this intial observation by showing that resveratrol is able to inhibit all 11 human HDAC enzymes and can even substantially alter the acetylation status in human tumor cells. Due to the manifold mechanisms of action that have been reported for resveratrol, we finally cannot determine the exact contribution of HDAC inihibition to the reduced proliferation of tumor cells. However, in previous studies of human derived hepatomas we could clearly demonstrate that HDAC inhibition can induce a profound antitumor effect *in vitro* [63] and *in vivo* [64]. Thus the described epigenetic modulating capacity of resveratrol adds another attractive antitumor mechanism to a substance with a quite low toxicity profile.

Beyond side effect profiles, issues of bioavailability are important in cancer drug development. For resveratrol a peak plasma concentration of approximately 2 µM with a rapid decline was reported [65]–[67]. At this low concentration we did not monitor an antiproliferative activity on our tumor cells. According to the low plasma concentrations achieved *in vivo*, resveratrol in its present form might be too weak for a single treatment application against solid tumors but could harbor tumor protecting longterm properties.

## Conclusions

Taken together, the described novel pan-HDACi activity of resveratrol opens up new perspectives for the clinical use as a chemopreventive drug or cancer therapeutic. The polyphenolic structure of resveratrol allows different modifications of the parental chemical structure enabling to develop new resveratrol analogs in order to improve bioavailability and/or HDACi activity. A resulting potent anticancer compound could combine several completely different mechanisms to hit cancer cells, which could be a way to avoid the development of therapy resistance.

## Supporting Information

File S1 Figure S1. Chemical structure of resveratrol. Figure S2. Differential cell response within the first 24 h after start of resveratrol treatment. Figure S3. No change of the acetylation status in Hep3B and HuH7 cells after resveratrol treatment. Figure S4. No resveratrol mediated hyperacetylation of histone protein H3 in Hep3B and HuH7 cells. Figure S5. Influence of SIRT1 activation by resveratrol in the HepG2 hepatoma cell context. Table S1. Calculated GoldScore values for resveratrol. Table S2. Calculated inhibitory concentrations (IC50) of resveratrol.
(PDF)
