# Correction: Resveratrol as a Pan-HDAC Inhibitor Alters the Acetylation Status of Jistone Proteins in Human-Derived Hepatoblastoma Cells

**DOI:** 10.1371/annotation/5b9a8614-1009-40ca-b90b-db817fe445c9

**Published:** 2013-09-04

**Authors:** Sascha Venturelli, Alexander Berger, Alexander Böcker, Christian Busch, Timo Weiland, Seema Noor, Christian Leischner, Sabine Schleicher, Mascha Mayer, Thomas S. Weiss, Stephan C. Bischoff, Ulrich M. Lauer, Michael Bitzer

There was an error in the article title.

The correct title is: Resveratrol as a Pan-HDAC Inhibitor Alters the Acetylation Status of Histone Proteins in Human-Derived Hepatoblastoma Cells

The correct citation is: Venturelli S, Berger A, Böcker A, Busch C, Weiland T, et al. (2013) Resveratrol as a Pan-HDAC Inhibitor Alters the Acetylation Status of Histone Proteins in Human-Derived Hepatoblastoma Cells. PLoS ONE 8(8): e73097. doi:10.1371/journal.pone.0073097 

