# Supplementary material for: Resveratrol as a Pan-HDAC Inhibitor Alters the Acetylation Status of Jistone Proteins in Human-Derived Hepatoblastoma Cells
Source: PLoS One. 2013 Aug 30;8(8):e73097. doi: 10.1371/journal.pone.0073097 (PMC3758278; doi:10.1371/journal.pone.0073097)
Supplement: File S1 — Figure S1. Chemical structure of resveratrol. Figure S2. Differential cell response within the first 24 h after start of resveratrol treatment. Figure S3. No change of the acetylation status in Hep3B and HuH7 cells after resveratrol treatment. Figure S4. No resveratrol mediated hyperacetylation of histone protein H3 in Hep3B and HuH7 cells. Figure S5. Influence of SIRT1 activation by resveratrol in the HepG2 hepatoma cell context. Table S1. Calculated GoldScore values for resveratrol. Table S2. Calculated inhibitory concentrations (IC50) of resveratrol. (PDF) [file pone.0073097.s001.pdf]

Supplementary Material

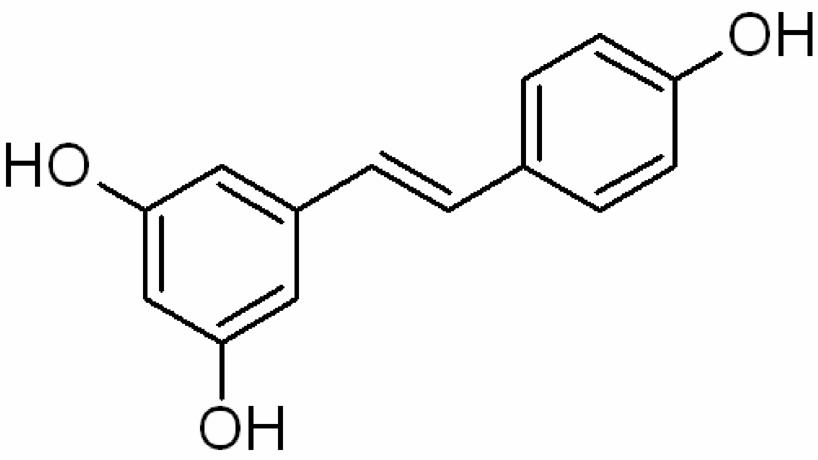

Supplementary Figure S1: Chemical structure of resveratrol. The polyphenolic alcohol resveratrol belongs to the group of phytoalexins and is mainly expressed in grapes. Shown is resveratrol in trans conformation.

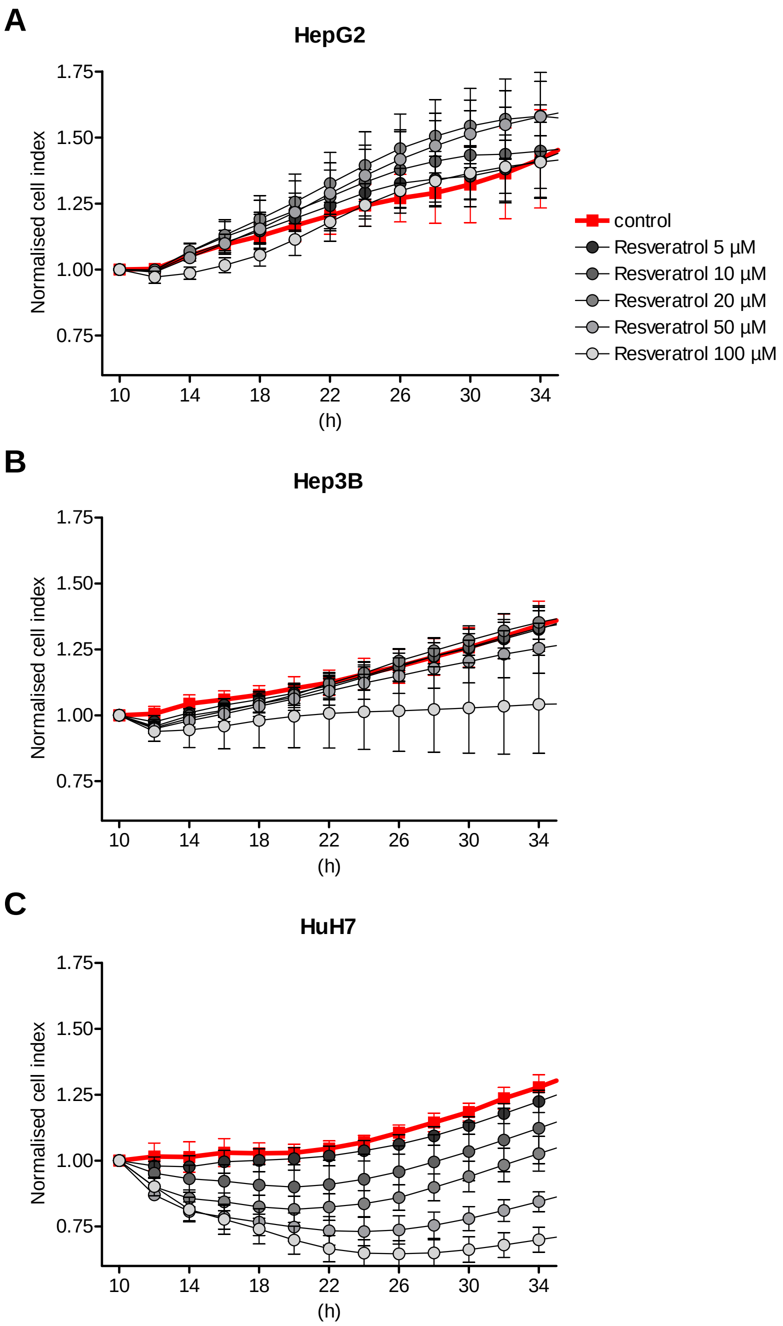

Supplementary Figure S2: Differential cell response within the first 24 h after start of resveratrol treatment. (A-C) HepG2 (A), Hep3B (B) and HuH7 (C) hepatoma tumor cells were treated with different concentrations of resveratrol (5 µM, 10 µM, 20 µM, 50 µM and 100 µM) or solvent as control. Here the timeframe between 10 h and 34 h of the displayed curves in Figure 3 were enlarged to illustrate the different reaction patterns. Curves of solvent control are highlighted in red. Cellular impedance was measured by the xCELLigence^{TM} SP system and calculated by the RTCA Software 1.2.1.1002. All cell index (CI) values were normalized when treatment started.

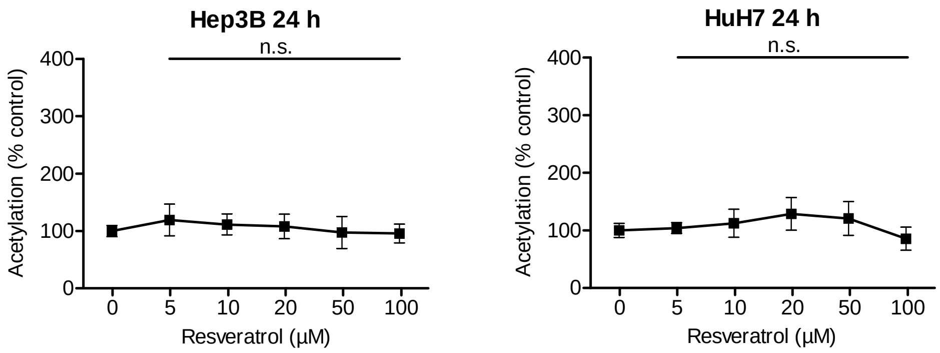

Supplementary Figure S3: No change of the acetylation status in Hep3B and HuH7 cells after resveratrol treatment. Determination of the overall acetylation of intracellular proteins in Hep3B and HuH7 hepatoma cells after incubation with various concentrations of resveratrol (5 µM, 10 µM, 20 µM, 50 µM and 100 µM) for 24 h. Bars represent mean ± SD of three independent experiments, each performed in triplicates; One-way ANOVA Dunnett´s multiple comparison test, n.s. indicates not significant.

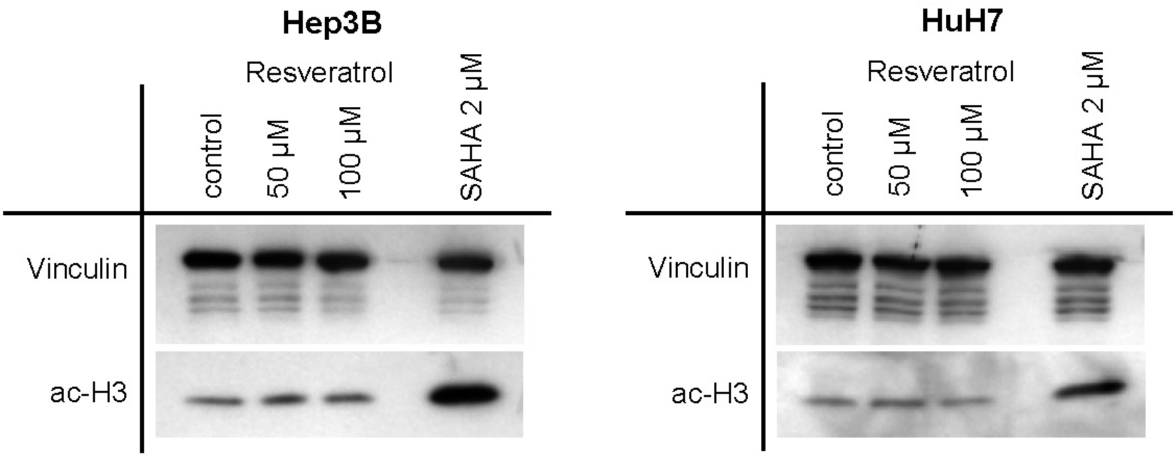

Supplementary Figure S4: No resveratrol mediated hyperacetylation of histone protein H3 in Hep3B and HuH7 cells. Western blot analyses of acetylated histone protein H3 revealed no hyperacetylation within Hep3B and HuH7 cells after 1 h of treatment with 50 µM and 100 µM resveratrol. As positive control 2 µM SAHA was used.

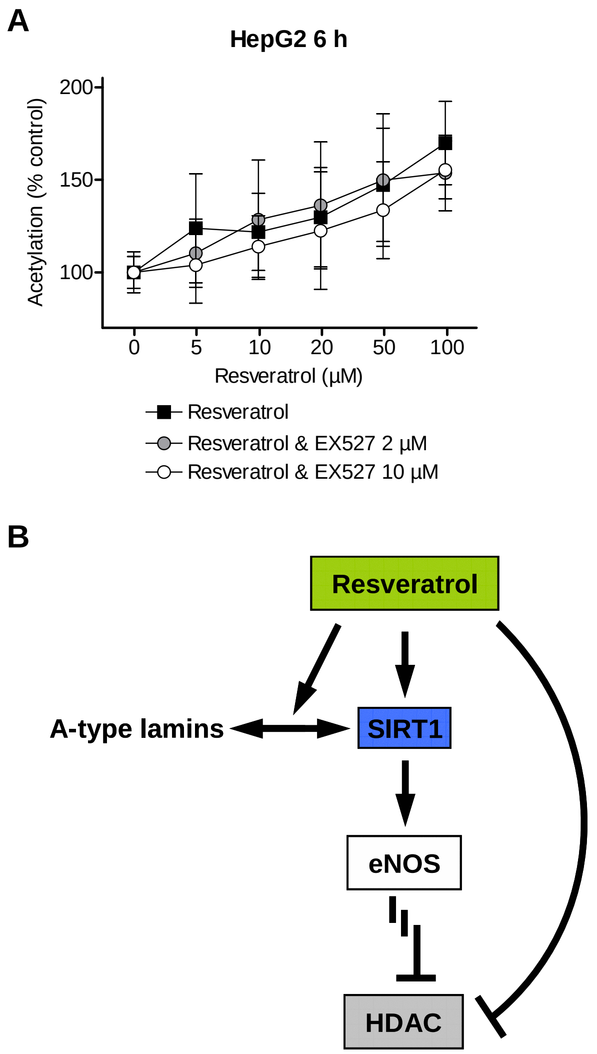

Supplementary Figure S5: Influence of SIRT1 activation by resveratrol in the HepG2 hepatoma cell context. (A) The impact of SIRT1 in the HepG2 system was analyzed determining global protein acetylation. Therefore HepG2 cells were incubated with the SIRT1 inhibitor EX527 in combination with resveratrol. Shown are mean of three independent experiments performed in triplicates ± SD. (B) Scheme of different possible interactions between resveratrol and HDACs. Due to the pleiotropic character of resveratrol several mechanisms by which resveratrol could modify HDAC activity are possible, e.g. resveratrol could inhibit HDACs via eNOS after activation of SIRT1, as reported in a wound healing model [57] or by lamin A mediated SIRT1 activation in a transgenic mouse model with a defect in A-type lamin maturation [56], and according to the presented *in silico* and enzyme based *in vitro* HDAC profiling data, also by a direct inhibition of classical HDACs.

Supplementary Table S1: Calculated GoldScore values for resveratrol. On basis of the *in silico* docking analysis GoldScore values were calculated for resveratrol using GOLD (version 4.1.2).

HDACi	HDAC2	HDAC4	HDAC7	HDAC8
**Resveratrol**	58.1	42.4	44.1	50.6

Supplementary Table S2: Calculated inhibitory concentrations (IC_{50}) of resveratrol. On basis of the real-time cell monitoring of HepG2, Hep3B and HuH7 hepatoma cells treated with resveratrol, the IC_{50} concentrations were calculated by the RTCA Software 1.2.1.1002 of the xCELLigence^{TM} SP system. Shown are mean ± SD of three independent experiments, each performed in triplicates; n.d. indicates not determinable.

Cell line	24 h	48 h	72 h	96 h
**HepG2**	n.d.	32.48±14.58 µM	33.3±16.04 µM	34.57±17.43 µM
**Hep3B**	n.d.	200.46±84.17 µM	52.67±9.98 µM	43.68±2.52µM
**HuH7**	44.06±18.97µM	28.9±17.07µM	27.59±15.31µM	51.14±24.87µM
